# *Xist* and *Tsix* Transcription Dynamics Is Regulated by the X-to-Autosome Ratio and Semistable Transcriptional States

**DOI:** 10.1128/MCB.00183-16

**Published:** 2016-10-13

**Authors:** Friedemann Loos, Cheryl Maduro, Agnese Loda, Johannes Lehmann, Gert-Jan Kremers, Derk ten Berge, J. Anton Grootegoed, Joost Gribnau

**Affiliations:** a Department of Developmental Biology, Erasmus MC, University Medical Center, Rotterdam, The Netherlands; b Erasmus MC Stem Cell Institute, Erasmus MC, University Medical Center, Rotterdam, The Netherlands; c Optical Imaging Center, Erasmus MC, University Medical Center, Rotterdam, The Netherlands

## Abstract

In female mammals, X chromosome inactivation (XCI) is a key process in the control of gene dosage compensation between X-linked genes and autosomes. *Xist* and *Tsix*, two overlapping antisense-transcribed noncoding genes, are central elements of the X inactivation center (*Xic*) regulating XCI. *Xist* upregulation results in the coating of the entire X chromosome by Xist RNA in *cis*, whereas *Tsix* transcription acts as a negative regulator of *Xist*. Here, we generated *Xist* and *Tsix* reporter mouse embryonic stem (ES) cell lines to study the genetic and dynamic regulation of these genes upon differentiation. Our results revealed mutually antagonistic roles for *Tsix* on *Xist* and vice versa and indicate the presence of semistable transcriptional states of the *Xic* locus predicting the outcome of XCI. These transcriptional states are instructed by the X-to-autosome ratio, directed by regulators of XCI, and can be modulated by tissue culture conditions.

## INTRODUCTION

Early during mammalian development, one of the two X chromosomes in female cells is transcriptionally inactivated. This X chromosome inactivation (XCI) process is initiated early during development and is then clonally propagated through a near-infinite number of cell divisions. Two X-linked noncoding genes, *Xist* and *Tsix*, play a key role in the regulation of XCI in mouse. *Xist* expression is upregulated on the future inactive X chromosome (Xi) (1, 2), and *cis* spreading of Xist leads to the recruitment of chromatin remodeling complexes that render X inactive (3, 4). *Tsix* is transcribed antisense to *Xist* and fully overlaps *Xist* (5). *Tsix* transcription and/or the produced Tsix RNA is involved in the repression of *Xist*, which includes Tsix-mediated chromatin changes at the *Xist* promoter (6–9).

*Xist* and *Tsix* are key components of *Xic*, the master switch locus that is regulated by XCI activators and inhibitors of XCI. XCI activators activate *Xist* and/or repress *Tsix*, whereas XCI inhibitors are involved in the repression of *Xist* and/or the activation of *Tsix*. In recent years, several XCI inhibitors have been described, including the pluripotency factors NANOG, SOX2, OCT4, REX1, and PRDM14, which provide a direct link between cell differentiation and initiation of XCI (10–13). These factors and other ubiquitously expressed XCI inhibitors, including CTCF (14, 15), repress the initiation of XCI through binding to multiple gene regulatory elements of *Xist* and *Tsix*. Genetic studies indicate that several of these elements might fulfill redundant roles in the regulation of XCI (16–18).

The X-linked gene *Rnf12* encodes a potent XCI activator, as the overexpression of *Rnf12* results in the ectopic initiation of XCI in differentiating transgenic embryonic stem (ES) cells (19). The encoded protein, RNF12, is an E3 ubiquitin ligase, which targets the XCI inhibitor REX1 for degradation (20). Degradation of REX1 by RNF12 is dose dependent, and 2-fold expression of RNF12 in female cells prior to XCI is important for female-specific initiation of this process. Chromatin immunoprecipitation sequencing (ChIP-seq) studies indicated REX1 binding in both *Xist* and *Tsix* regulatory regions. REX1-mediated repression of *Xist* involves indirect mechanisms, including the activation of *Tsix*, as well as the direct regulation of *Xist* by a competition mechanism, where REX1 and YY1 compete for shared binding sites in the F repeat region in *Xist* exon 1 (21).

*Rnf12* knockout studies revealed a reduction of XCI in differentiating female *Rnf12*^+/−^ ES cells and a near loss of XCI initiation in *Rnf12*^−/−^ ES cells (16). However, the remaining initiation of XCI in a subpopulation of *Rnf12*^+/−^ cells also indicates the presence of additional XCI activators, as XCI is not initiated in male cells. This is supported by *in vivo* studies revealing that mice with a conditional deletion of *Rnf12* in the developing epiblast are born alive (22). *Jpx* and *Ftx* have been described as putative XCI activators (15, 23, 24). Both genes are located in a region 10 to 100 kb distal to *Xist*, and knockout studies indicated that both genes are involved in *Xist* activation. Although transgene studies implicated that *Jpx* is a *trans* activator of *Xist*, recent studies involving a knockout of a region from *Xite* up to the *Xpr* region did not reveal a *trans* effect, suggesting that the predominant function of *Ftx* and *Jpx* in XCI is the *cis* activation of *Xist* (25).

Interestingly, examination of the higher-order chromatin structure revealed that *Xist* and *Tsix* are located in two distinct neighboring topologically associated domains (TADs) (26, 27). Positive regulators of *Xist*, including *Jpx* and *Ftx*, are located in the same TAD. Similarly, the *Tsix*-positive regulators *Xite*, *Tsx*, and *Linx* are located in the *Tsix* TAD, suggesting that these two TADs represent the minimal X inactivation center covering all *cis*-regulatory elements, which are regulated by *trans*-acting XCI activators and inhibitors (27–29). During development or ES cell differentiation, the XCI activator concentration in female cells is 2-fold higher than that in male cells, which is sufficient to direct female-exclusive initiation of XCI. Stochastic initiation of XCI and rapid feedback mechanisms, including the shutdown of *Tsix*, *Rnf12*, and other XCI activators in *cis*, direct a highly efficient XCI process facilitated by the requirement of a loss of pluripotency for the initiation of XCI (30).

The overlapping gene bodies of *Xist* and *Tsix* and the mutually antagonistic roles of these two genes hamper clear insights in the regulatory mechanisms that govern *Xist* and *Tsix* transcription. To be able to study the independent pathways directing *Xist* and *Tsix* transcription, we have generated *Xist* and *Tsix* reporter alleles, with fluorescent reporters replacing the first exon of *Xist* and/or *Tsix*. Our studies indicate antagonistic roles for both *Xist* and *Tsix* and show that RNF12 and REX1 regulate XCI through both the repression of *Tsix* and the activation of *Xist*. Live-cell imaging confirms a reciprocal correlation of *Xist* and *Tsix* transcription but also reveals that their regulation is not strictly concerted and rather stable in time. Interestingly, the loss of an X chromosome severely affects the dynamics of both *Xist* and *Tsix* expression and results in two different cell populations with semistable transcriptional states, which are absent in female ES cells. This indicates a regulatory role for the X-to-A ratio regarding the nuclear concentration of X-encoded *trans*-acting factors. Similar semistable transcriptional states are observed in female ES cells grown in medium supplemented with MEK and glycogen synthase kinase 3 (GSK3) inhibitors, displaying distinct XCI characteristics upon ES cell differentiation. Our findings suggest that XCI activators are required to install a uniform transcriptional state of the *Xic* locus that allows the proper upregulation of *Xist* upon ES cell differentiation.

## MATERIALS AND METHODS

### Plasmids and antibodies.

Plasmids used for the generation of transgenic cell lines were pCAG-Rex1-Flag, pCAG-Rnf12-Flag (20), and pCAG-mTagBFP2-Ezh2-Flag. The coding sequence of mTagBFP2 was inserted N terminally to the EZH2 coding sequence amplified from mouse cDNA and cloned into pCAG-Flag to generate pCAG-mTagBFP2-Ezh2-Flag. Antibodies used were those against Flag-M2 (Sigma), REX1 (Abcam and Santa Cruz), RNF12 (Abnova), H3K27me3 (Diagenode), and CD31-fluorescein isothiocyanate (FITC) (BD Biosciences).

### Cell lines.

Standard ES cell culture conditions included serum plus leukemia inhibitory factor (LIF), and both ES cell and differentiation conditions were described previously (16). 2i+LIF conditions were Dulbecco's modified Eagle's medium (DMEM) supplemented with 100 U/ml penicillin-streptomycin, 20% KnockOut serum replacement (Gibco), 0.1 mM nonessential amino acids (NEAA), 0.1 mM 2-mercaptoethanol, 5000 U/ml LIF, 1 μM the MEK inhibitor PD0325901 (Stemgent), and 3 μM the GSK3 inhibitor CH99021 (Stemgent). Transgenic ES cell lines were generated by using the wild-type female line F1 2-1 (129/Sv-Cast/Ei) and the wild-type male line J1 (129/Sv). A bacterial artificial chromosome (BAC) targeting strategy was used as described previously (31). In short, the Xist knock-in was created as follows: an enhanced green fluorescent protein (EGFP)-neomycin resistance cassette flanked by *lox* sites was targeted by homologous recombination in bacteria to a BAC (31). 5′- and 3′-targeting arms were amplified from a BAC by using primers 1 and 2 and primers 5 and 6, respectively. With the modified BAC, wild-type ES cells were targeted, and the resistance cassette was removed by transient Cre transfection, resulting in the ES cell line Xist-GFP. An Xist ScrFI restriction fragment length polymorphism (RFLP) with primers 4 and 20 was used to screen drug-resistant clones for correct recombination events. The Tsix knock-in was created as follows: an mCherry-neomycin resistance cassette flanked by *lox* sites was targeted by homologous recombination in bacteria to a BAC. 5′- and 3′-targeting arms were amplified from a BAC by using primers 25 and 27 and primers 29 and 30, respectively. With the modified BAC, wild-type or Xist-GFP ES cells were targeted, and resistance cassettes were removed by transient Cre transfection, resulting in the cell lines Tsix-CHERRY and Xist-GFP/Tsix-CHERRY, respectively. A Tsix PCR-length polymorphism with primers 36 and 41 was used to screen drug-resistant clones for correct recombination events. Rex1, Rnf12, and mTagBFP2-Ezh2 transgenes were introduced by electroporation (Gene Pulser Xcell; Bio-Rad) and subsequent puromycin selection. Overexpression of transgenes was verified by Western blotting and reverse transcription-quantitative PCR (RT-qPCR). The XGTC-XO ES cell line was generated by subcloning Xist-GFP/Tsix-CHERRY cells via single-cell sorting on a FACSAria III platform. Single-cell-derived subclones were screened for the loss of the wild-type X chromosome by a Pf1MI RFLP located in the X-linked gene *Atrx* using primers 68 and 69.

### Fluorescence-activated cell sorter (FACS) analysis and cell sorting.

Single-cell suspensions were prepared by trypsin-EDTA treatment for 7 min at 37°C. Duplets were excluded by appropriate gating, and dead/dying cells were excluded by Hoechst 33258 staining (1 μg/ml; Molecular Probes). Relative fluorescence intensities were determined for EGFP and mCherry. Cell analysis was performed on an LSRFortessa instrument, and cell sorting was performed on a FACSAria III instrument (BD Biosciences) with FACSDiva software. Statistical analysis was performed with FlowJo.

### Expression analysis.

RNA was isolated by using TRIzol reagent (Invitrogen) according to the manufacturer's instructions. DNase I treatment was performed to remove genomic DNA, and cDNA was prepared by using random hexamers and SuperScript II (Invitrogen). RT-qPCR was performed with a CFX384 real-time PCR detection system (Bio-Rad) using Fast SYBR green master mix (Applied Biosystems) and primers described in Table S1 in the supplemental material. Results were normalized to values for actin by using the Δ*C_T_* method and mostly represented as fold changes versus values for day 0 of differentiation.

### Live-cell imaging and image analysis.

Cells were preplated to remove feeders, and differentiation was initiated 12 h prior to the start of imaging. Cells were seeded at a low density (10^4^ cells/well) in a 6-well glass-bottom dish (catalog number P06G-1.5-20-F; MatTek) coated with human plasma fibronectin (Millipore). Imaging was performed on a Leica SP5 AOBS at 37°C with 5% CO_2_ using adaptive focus control to keep cells in focus during the entire experiment. Pictures were taken every 20 min for a total of 68 h. Tiled images were acquired and automatically stitched to record a large field of view at a sufficient resolution to resolve subcellular structures and monitor cells over time. An average projection of z-stacks was generated in Fiji (version 1.45b), and background-corrected integrated fluorescence intensities for EGFP and mCherry were measured for single cells over the entire time frame that a given cell was clearly discriminable. Based on recorded values, linear regression by the least-squares method was performed to calculate the straight line that best fit the data. The slope of this function with fluorescence intensity (FI) being dependent on time was used as a proxy for *Xist* or *Tsix* promoter activity. The threshold for *Xist* activation was calculated by using 3.29 standard deviations (corresponding to 99.9% within the confidence interval) of mean EGFP FI values measured in cells within the first 6 h of the time-lapse experiment.

### Fluorescent *in situ* hybridization and immunofluorescence.

For Xist/Tsix RNA fluorescent *in situ* hybridization (FISH) and immunofluorescence stainings, cells were grown on or absorbed to polylysinated coverslips. For RNA FISH, cells were fixed for 10 min with 4% paraformaldehyde (PFA)–phosphate-buffered saline (PBS) at room temperature, washed with 70% ethanol (EtOH), permeabilized for 4 min with 0.2% pepsin at 37°C, and postfixed with 4% PFA–PBS for 5 min at room temperature. Coverslips were washed twice with PBS and dehydrated in a gradient of 70%, 90%, and 100% EtOH. Nick-labeled DNA probes (digoxigenin for the Xist/Tsix probe and biotin for the Tsix probe) were dissolved in a hybridization mixture (50% formamide, 2× SSC [1× SSC is 0.15 M NaCl plus 0.015 M sodium citrate], 50 mM phosphate buffer [pH 7.0], 10% dextran sulfate) and 100 ng/μl mouse Cot DNA to a final concentration of 1 ng/μl. The probe was denatured for 5 min and prehybridized for 45 min at 37°C, and coverslips were incubated overnight in a humid chamber at 37°C. After hybridization, coverslips were washed once in 2× SSC, three times in 50% formamide–2× SSC (both at 37°C), and twice in TST (0.1 M Tris, 0.15 M NaCl, 0.05% Tween 20) at room temperature. Blocking was done in bovine serum albumin (BSA)-TST for 30 min at room temperature. Detection was done by subsequent steps of incubation with antidigoxigenin (Boehringer) and two FITC-labeled antibodies for Xist/Tsix RNA detection or with antibiotin (Roche) and two rhodamine-labeled antibodies for Tsix RNA detection in blocking buffer for 30 min at room temperature. Coverslips were washed twice with TST between detection steps and once finally with TS (0.1 M Tris, 0.15 M NaCl). Dehydrated coverslips were mounted with ProLong Gold Antifade with 4′,6-diamidino-2-phenylindole (DAPI) (Molecular Probes). For immunofluorescence, cells were fixed for 10 min at room temperature in 4% PFA–PBS, followed by three washes in PBS and permeabilization in 0.25% Triton X-100–PBS. Blocking was done in blocking solution (0.5% BSA plus 1% Tween 20 in PBS) for 1 h at room temperature. All antibody incubation steps were done for 1 h at room temperature in blocking solution, followed by three washes in blocking solution. The following primary antibodies were used: anti-Flag-M2 (1:1,000) and anti-H3K27me3 (1:500). Secondary antibodies used were conjugated to Alexa Fluor 488 or Alexa Fluor 546 (1:500; Molecular Probes).

### RNA sequencing.

RNA samples were collected 2 days after FACS analysis of different populations of undifferentiated ES cells, prepared with the TruSeq RNA kit, sequenced according to the Illumina TruSeq v3 protocol on a HiSeq2000 instrument with a single read of 43 bp and a 7-bp index, and mapped against the mouse mm10/GRCm38 reference genome by using TopHat (version 2.0.10). Gene expression values were called by using Cufflinks (version 2.1.1).

### Statistical methods.

A confidence interval of 95% was calculated as $p-[1.96\sqrt{\frac{p(1-p)}{n}}]$ to $p+[1.96\sqrt{\frac{p(1-p)}{n}}]$, where *n* is the number of cells counted and *p* is the percentage of Xist clouds scored.

The standard deviation was calculated as $\sqrt{\frac{\Sigma (x-\overset{¯}{x}{)}^{2}}{n}}$, where *x* is the sample mean and *n* is the sample size.

Linear regression was performed by using the least-squares method.

The Pearson product-moment correlation coefficient was calculated as

$$r=\frac{\Sigma (x-\overset{¯}{x})(y-\overset{¯}{y})}{\sqrt{\Sigma (x-\overset{¯}{x}{)}^{2}\;\Sigma (y-\overset{¯}{y}{)}^{2}}}$$

where *x* and *y* are values of paired data.

Single-factor analysis of variance (ANOVA) using the *F* distribution was used to test the null hypothesis that all of three or more groups of samples belong to populations with the same mean values.

The chi-square test of independence was used to test if the observed frequencies for three or more groups are equal to the expected frequencies, calculated as

$$x^{2}=\Sigma_{i}\;\Sigma_{j}\;\frac{(O_{ij}-E_{ij}{)}^{2}}{E_{ij}}$$

where *O_ij_* is the observed frequency and *E_ij_* is the expected frequency.

Statistical significance as determined by the two-proportion *z* test was calculated as

z=p^1−p^2p¯(1−p¯)(1n1+1n2)

where *n* is the number of cells analyzed and *p̂* and *p̄* correspond to the proportion and average proportion, respectively.

## RESULTS

### Antagonistic roles for *Xist* and *Tsix*.

**FIG 1:**
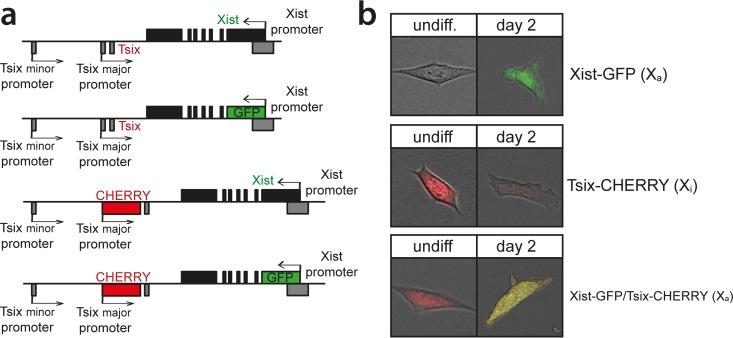
Generation of the reporter alleles. (a) Map of the *Xist*/*Tsix* locus showing the design of the reporter cell lines. (b) Exemplary pictures of undifferentiated and differentiated cells.

**FIG 2:**
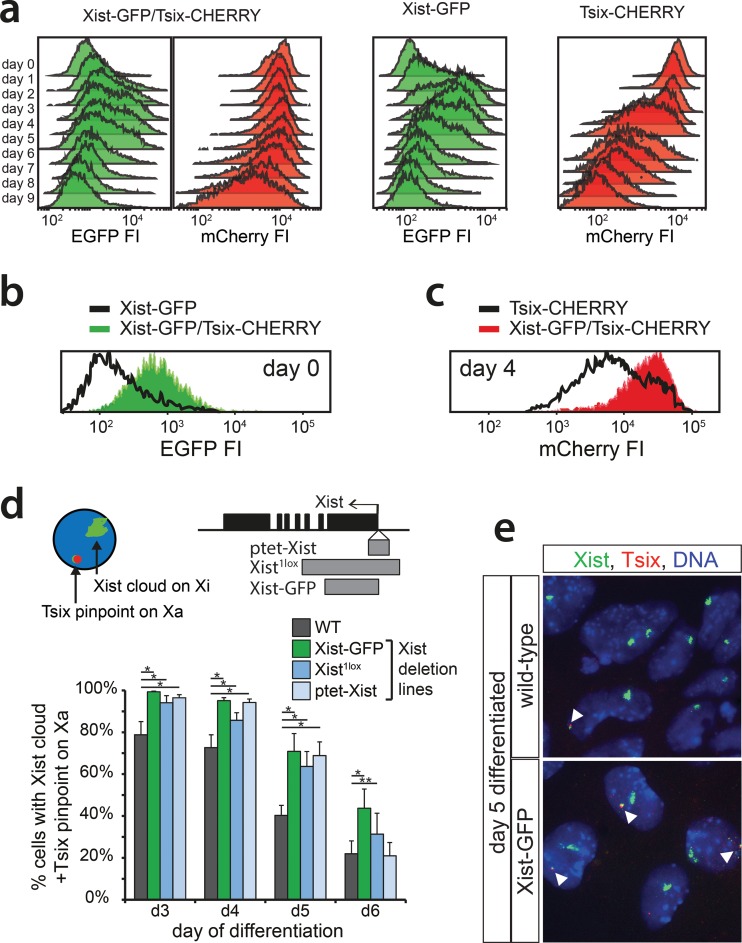
*Xist* and *Tsix* reporter lines reveal antagonistic roles for *Xist* and *Tsix*. (a) Histograms of EGFP and mCherry FI distributions as determined by FACS analysis. Days 0 through 9 of differentiation are depicted for Xist-GFP, Tsix-CHERRY, and Xist-GFP/Tsix-CHERRY cells. (b and c) Histograms of EGFP (b) and mCherry (c) FI distributions as determined by FACS analysis. Black outlines represent the undifferentiated Xist-GFP single-knock-in cell line (b) and the Tsix-CHERRY single-knock-in cell line at day 4 of differentiation (c). Solid colors represent FI distributions for Xist-GFP/Tsix-CHERRY. (d) Quantification of two-color RNA FISH to detect Xist and Tsix transcripts at different time points of differentiation. The proportion of cells with an Xist cloud, identifying Xi and a Tsix pinpoint from Xa, is shown. The top right panel shows the exon-intron structure of *Xist*, and gray bars indicate the deleted region of the respective deletion lines. Error bars indicate 95% confidence intervals (*n* > 150 for all time points and cell lines). Asterisks indicate a *P* value of <0.05 (*) or a *P* value of <0.1 (**) by a two-proportion *z* test. (e) Xist/Tsix two-color RNA FISH of wild-type and Xist-GFP cells. The green probe detects Xist and Tsix, and the red probe detects only Tsix. Xi is identified by the presence of a Xist cloud, and Tsix transcription from Xa is indicated by the presence of a separate two-color pinpoint in the same nucleus.

X chromosome inactivation (XCI) is orchestrated by *Xist* and *Tsix*, two noncoding RNA genes with antagonistic roles. *Xist* is essential for XCI to occur in *cis* (32, 33), while *Tsix* is a negative regulator of XCI (6, 34). Analysis of the regulation of *Xist* and *Tsix* and their relationship during the onset of XCI is hampered by the architecture of the locus. *Tsix* entirely overlaps *Xist* and is transcribed in an antisense direction, and manipulation of one of the two genes always affects the antisense partner. To be able to monitor and manipulate the activities of the *Xist* and *Tsix* promoters independently, we generated a series of reporter lines in murine ES cells (Fig. 1a). Exploiting BAC-mediated homologous recombination in polymorphic female 129/Sv-Cast/Ei ES cells (31), exons 1 of *Xist* and *Tsix*, located on the Cast/Eij X chromosome, were replaced with EGFP and mCherry coding sequences, respectively (see Fig. S1 and S2 in the supplemental material). The expression of the reporters was thus controlled by the endogenous promoters of these two noncoding genes (Fig. 1b). Wild-type female 129/Sv-Cast/Ei ES cells show preferential inactivation of the 129/Sv X chromosome in 70% of the cells, attributed to single-nucleotide polymorphisms (SNPs) in regulatory elements that affect the regulation of *Xist* and *Tsix* throughout the ES cell differentiation process. We found that the alleles behaved as full *Xist*/*Tsix* knockouts, resulting in a complete skewing of XCI, because splice donor sites at the 3′ ends of the targeted exons were removed, and poly(A) signals downstream of the reporters terminated transcription (see Fig. S1 and S2 in the supplemental material). By successive rounds of targeting followed by Cre-mediated removal of selection markers, three ES cell lines were obtained: (i) Xist promoter-EGFP knock-in (Xist-GFP) cells, (ii) Tsix promoter-mCherry knock-in (Tsix-CHERRY) cells, and (iii) cells with a double knock-in on the same allele with the Xist promoter-EGFP and the Tsix promoter-mCherry (Xist-GFP/Tsix-CHERRY). Differentiation of these lines and expression of *Xist* and *Tsix* on the remaining wild-type 129/Sv allele were unperturbed (see Fig. S3a in the supplemental material). Xist-GFP/Tsix-CHERRY cells displayed kinetics of Xist cloud formation similar to those of wild-type cells albeit with slightly reduced percentages. This is expected from a full *Xist* knockout, and probably due to stochastic initiation (see Fig. S3b in the supplemental material). FACS analysis of EGFP and mCherry expression for all three ES cell lines showed faithful recapitulation of the behavior of wild-type *Xist* and *Tsix* during the first days of differentiation (Fig. 2a), which was not delayed by a half-life for EGFP and mCherry that ranged from 11 to 14 h (see Fig. S3c in the supplemental material). As expected, comparison of Xist-GFP/Tsix-CHERRY ES cells, which allows independent tracking of *Xist*/*Tsix*, with Xist-GFP ES cells shows EGFP derepression in undifferentiated cells when *Tsix* is deleted in *cis* (Fig. 2b). Comparison of Xist-GFP/Tsix-CHERRY with Tsix-CHERRY revealed delayed downregulation of the mCherry reporter in the double-knock-in cell line (Fig. 2c), indicating a role for *Xist* in silencing of *Tsix*. The delay in mCherry downregulation cannot be attributed to differences in mCherry expression/*Tsix* promoter activity between Xi (in the Tsix-CHERRY line) and Xa (in the Xist-GFP/Tsix-CHERRY line), suggesting that *Tsix* downregulation on the future Xa chromosome is compromised upon ES cell differentiation in the absence of *Xist* (see Fig. S3a in the supplemental material). To verify that this effect is not due to the deletion of any DNA elements involved in the repression of *Tsix* in Tsix-CHERRY cells, we performed two-color RNA FISH to distinguish between Xist and Tsix transcripts in differentiating ES cells. Three independent *Xist* deletion lines, Xist-GFP, Xist^1lox^, and ptet-Xist, harboring an insertion of a doxycycline-inducible promoter replacing the endogenous Xist promoter (35; A. Loda, unpublished data), show persisting *Tsix* transcription from Xa compared to that in wild-type cells (Fig. 2d and e). Taken together, these results show that *Xist* and *Tsix* display antagonistic roles, directly influencing the expression levels of each other on Xa during the early phases of ES cell differentiation. These results also highlight the need to investigate the dynamics of their early genetic regulation on the uncoupled allele in Xist-GFP/Tsix-CHERRY cells.

### Dynamics of regulation of the *Xic* locus by live-cell imaging.

**FIG 3:**
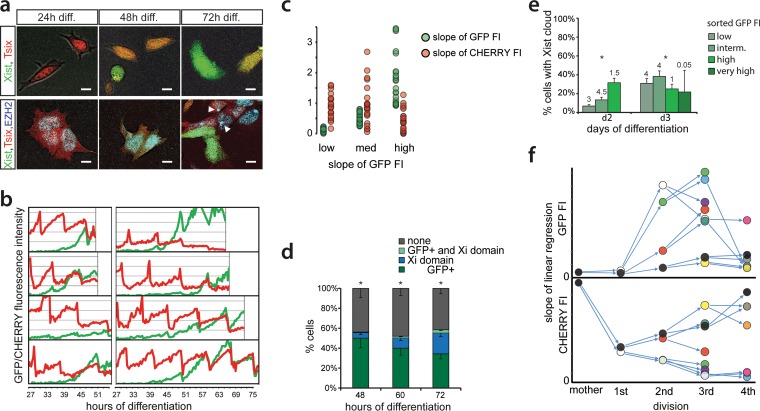
Time-lapse imaging of live cells. (a) Exemplary pictures of Xist-GFP/Tsix-CHERRY cells (top) and Xist-GFP/Tsix-CHERRY+mTagBFP2-Ezh2 cells (bottom) taken at different time points of differentiation during time-lapse imaging. Bars, 5 μm. (b) Whole-cell integrated FI values for EGFP (green) and mCherry (red) plotted over time for several exemplary cells during time-lapse imaging. (c) Linear regression of FI over time for each cell cycle. The slope of the linear regression as a proxy for promoter activity is plotted. Values for EGFP FIs are binned into low (lowest tercile), medium (intermediate tercile), and high (highest tercile), and the corresponding values for mCherry are plotted next to these values. (d) Quantification of the presence of the mTagBFP2-Ezh2 focus/Xi domain and/or high levels of EGFP at different time points of differentiation in Xist-GFP/Tsix-CHERRY+mTagBFP2-Ezh2 cells. Error bars indicate 95% confidence intervals (*n* = 162 for 48 h, *n* = 215 for 60 h, and *n* = 277 for 72 h). (e) Day 2 and day 3 differentiating Xist-GFP/Tsix-CHERRY cells were FACS sorted into EGFP-low, -intermediate, -high, and -very-high fractions. Graphs show quantification of Xist RNA FISH in these fractions. The number on top of each fraction represents the relative abundance within the population before sorting. Error bars indicate 95% confidence intervals (*n* > 250 for all time points and fractions). (f) Pedigree of an exemplary cell monitored through four cell divisions. (Top) The slope of the linear regression as described above for panel c is shown for EGFP FI. (Bottom) Slope of the linear regression for mCherry. The same-colored dots represent the same cell and thus indicate values for EGFP in the top panel and mCherry in the bottom panel. Arrows connecting dots indicate a mother cell-to-daughter cell relationship. Asterisks in panels d and e indicate a *P* value of <0.05, as calculated by a chi-square test.

To further analyze the dynamics of *Xist* and *Tsix* regulation, we performed live-cell imaging of differentiating Xist-GFP/Tsix-CHERRY cells for extended periods of time by confocal microscopy (Fig. 3a). The integrated EGFP and mCherry fluorescence intensities (FIs) of entire single cells were measured, resulting in semioscillating patterns due to the accumulation of fluorescent reporters followed by dilution upon cell division (Fig. 3b). For each cell cycle, the slope of the linear regression of the integrated FI over time gives an estimate of the activity of the *Xist* and *Tsix* promoters. Binning cell cycles with low, medium, and high increases in EGFP FI into groups and comparing the corresponding values for mCherry confirm a concerted anticorrelated regulation independent of antisense transcription, with EGFP being upregulated before downregulation of mCherry (Fig. 3c). Next, we set a threshold for the mean EGFP FI to estimate at which point the EGFP FI rises above background noise. Low values for the slope of mCherry before and high values after *Xist* activation argue that in spite of concomitant anticorrelated regulation, *Xist* and *Tsix* are independently and stochastically regulated (see Fig. S4a in the supplemental material).

To unravel the relationship between the activation of *Xist* and *Tsix* and the establishment of Xi, we introduced an mTagBFP2-Ezh2 fusion gene into Xist-GFP/Tsix-CHERRY ES cells (see Fig. S4b and c in the supplemental material). Since we were not able to continually monitor high numbers of cells until an Xi domain appeared, we instead scored cells at different time points of differentiation (Fig. 3d). The results show that high GFP levels almost never concur with an EZH2/Xi domain. RNA FISH analysis on day 2 differentiated FACS-sorted low-, intermediate-, high-, and very-high-EGFP cells but demonstrated that both *Xist* promoters become activated and that EGFP upregulation and XCI initiation correlate (Fig. 3e). At day 3, the EGFP^high^ and EGFP^veryhigh^ FACS-sorted fractions of cells contained less Xist clouds than did the EGFP^intermediate^ fraction. This suggests that EGFP^high^ and EGFP^veryhigh^ cells downregulate EGFP before Xist clouds become detectable but also indicates the presence of a subpopulation of cells that strongly and consistently activate Xist-GFP without upregulation of *Xist* on the wild-type X chromosome.

Live-cell imaging also enabled us to monitor single cells through mitosis and monitor the fate of daughter cells through successive rounds of cell division. Plotting of the slope of the EGFP/mCherry FI for each generation confirms the above-described anticorrelation of *Xist* and *Tsix* activity for each given cell (Fig. 3f). Moreover, daughter cells display strikingly similar patterns of *Xist* and *Tsix* promoter activities, indicating that they generally follow the same fate. This implies that switches of *Xist* and *Tsix* activity occur rarely or slowly and that once a certain transcriptional state is established, it is stably transmitted through cell division and relatively resistant to changes or reversal. Taken together, data from live-cell imaging and fate mapping suggest that on an uncoupled allele, *Xist* and *Tsix* are antagonistically regulated in a developmentally concerted manner, even though up- and downregulation of both genes *per se* are independent and probably stochastic.

### Effects of activators and inhibitors on XCI.

**FIG 4:**
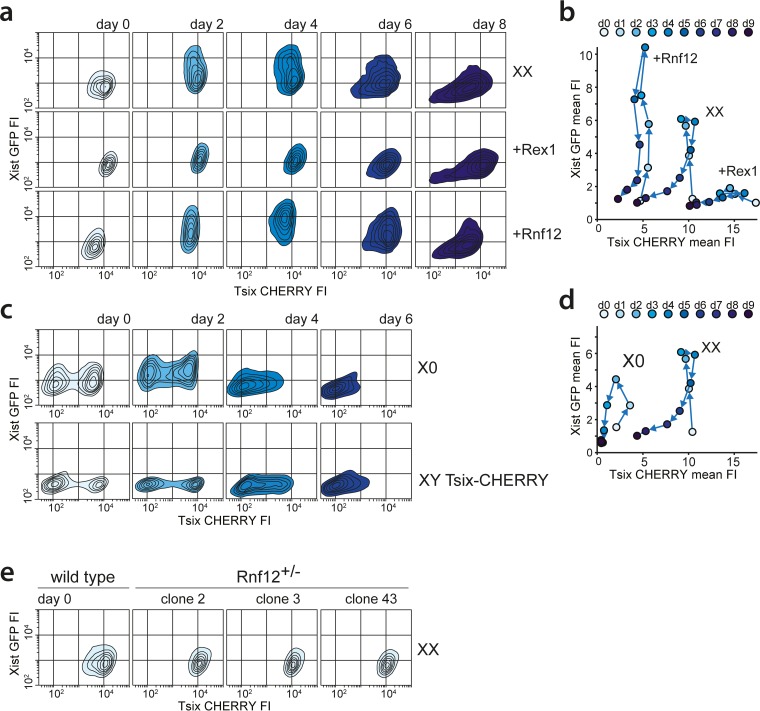
Impact of the RNF12-REX1 regulatory network on *Xic* regulation. (a) Contour plots data from FACS analysis showing EGFP and mCherry FIs at different time points of differentiation for Xist-GFP/Tsix-CHERRY (XX), Xist-GFP/Tsix-CHERRY+Rex1 (+Rex1), and Xist-GFP/Tsix-CHERRY+Rnf12 (+Rnf12) cells. For all experiments, 100,000 cells were analyzed per time point. Starting from the outermost contour, lines represent 7.5%, 22.5%, 37.5%, 52.5%, 67.5%, and 82.5% of the total events (logarithmic scale). (b) Same as panel a but with mean FIs for EGFP and mCherry plotted (linear scale). (c) Contour plots of data from FACS analysis showing EGFP and mCherry FIs at different time points of differentiation for the XGTC-XO (top) and XY Tsix-CHERRY (bottom) lines. Starting from the outermost contour, lines represent 7.5%, 22.5%, 37.5%, 52.5%, 67.5%, and 82.5% of the total events (logarithmic scale). (d) Same as panel d but with mean FIs for EGFP and mCherry plotted for the XGTC-XO line (linear scale). (e) Contour plots of data from FACS analysis showing EGFP and mCherry FIs in undifferentiated Xist-GFP/Tsix-CHERRY (XX) cells and for clones 2, 3, and 43 of Xist-GFP/Tsix-CHERRY Rnf12^+/−^ ES cell lines. Starting from the outermost contour, lines represent 7.5%, 22.5%, 37.5%, 52.5%, 67.5%, and 82.5% of the total events (logarithmic scale).

RNF12 functions as a *trans* activator of XCI (19) by targeting REX1, a repressor of XCI, for proteasomal degradation (20). Previous work indicated that REX1 might have a dual role in the activation of XCI by activating *Tsix* and repressing *Xist* (12, 16, 20). To dissect this XCI regulatory network and determine the role of these factors in the regulation of *Xist* and *Tsix* in ES cell lines harboring uncoupled *Xist/Tsix* alleles, we introduced Rnf12 and Rex1 transgenes into the three knock-in cell lines. Clones chosen for analysis consistently overexpressed *Rnf12* and *Rex1* by 2- to 3-fold compared to the levels in wild-type cells (see Fig. S5a in the supplemental material). FACS analysis of differentiating Xist-GFP/Tsix-CHERRY ES cells showed that Rnf12 and Rex1 transgenes had a clear effect on the EGFP and mCherry reporters (Fig. 4a and b). REX1 strongly repressed the *Xist* promoter and activated the *Tsix* promoter. Conversely, *Rnf12* overexpression resulted in increased activation of EGFP and reduced mCherry expression. This was also evident from quantitative analysis of RNA levels by qPCR. In the Xist-GFP/Tsix-CHERRY line, both *Xist* and EGFP were upregulated by an Rnf12 transgene and downregulated by a Rex1 transgene, while the opposite effect was observed for *Tsix* and mCherry (see Fig. S5b in the supplemental material). Since we monitored the uncoupled allele in a comparatively well-preserved genomic context, we can exclude any indirect effects due to interference from the corresponding antisense partner. In the presence of the antisense partner, in the single-knock-in Xist-GFP and Tsix-CHERRY lines, we observed that the effect of *Rnf12* and *Rex1* overexpression was strongly attenuated (see Fig. S5b in the supplemental material). This finding indicates that antisense transcription or the antisense transcript represses the transcription of the *Xist* and *Tsix* promoters located on the opposite strand and that a balanced allele might be necessary for the proper integration of regulatory signals.

The major difference between female cells that undergo XCI and male cells that do not is the X-to-A ratio. To better investigate the effects of changes in this X-to-A ratio on *Xist* and *Tsix* expression, we screened Xist-GFP/Tsix-CHERRY cells for subclones that had lost the wild-type 129 X chromosome by using an X-linked RFLP (see Fig. S5c in the supplemental material). These XO lines showed a stable karyotype (see Fig. S5d in the supplemental material), but comparison of these XO lines (XGTC-XO) with the XX Xist-GFP/Tsix-CHERRY double-knock-in ES cell line indicated that the dynamics of both GFP and mCherry expression during ES cell differentiation were severely affected by the loss of the wild-type X chromosome (Fig. 4c, top, and d). In addition, the XO cells were present in two distinct mCherry-high and mCherry-low populations. This bimodal mCherry distribution was also observed for the XY Tsix-CHERRY-only knock-in cells (Fig. 4c, bottom), indicating that the dynamics of these states is affected by the X-to-A ratio.

To test whether these effects are solely related to the *Rnf12* copy number, we generated three independent XX Xist-GFP/Tsix-CHERRY Rnf12^+/−^ heterozygous knockout cell lines where *Rnf12* was mutated on the 129/Sv allele (see Fig. S5e, g, and h in the supplemental material). Examination of these ES cell lines during differentiation shows a severe reduction in the upregulation of the Xist-GFP reporter allele (see Fig. S5i in the supplemental material). However, the two subpopulations found in undifferentiated XGTC-XO and Tsix-CHERRY-only ES cells are absent in Xist-GFP/Tsix-CHERRY Rnf12^+/−^ cells (Fig. 4e), which show a FACS profile similar to that of Rex1-transgenic Xist-GFP/Tsix-CHERRY cells. A decrease in *Rnf12* levels, therefore, does not explain the reduced mCherry expression level throughout ES cell differentiation observed in XGTC-XO ES cells. In addition, comparison of *Tsix* RNA expression levels in male and female ES cell lines by qPCR analysis confirmed that lower levels of *Tsix* RNA are present in male ES cells (see Fig. S5f in the supplemental material). These findings indicate that more X-encoded factors are involved in the regulation of XCI and that the X-to-A ratio also directs the dose-dependent activation of *Tsix*.

### Semistable transcriptional states of the *Xic* locus predict outcome of XCI.

**FIG 5:**
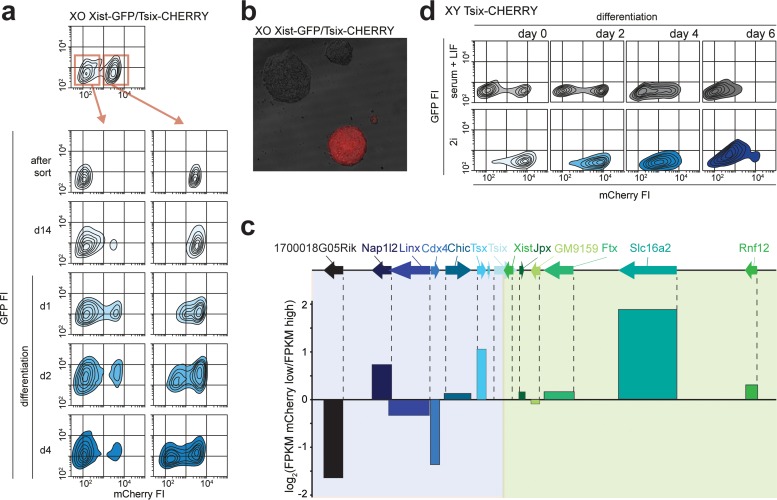
Two stable states of *Xic* in XO cells. (a) Contour plots of data from FACS analysis showing EGFP and mCherry FIs for the XGTC-XO line. The top panel depicts the original population with a bimodal mCherry distribution, and at the bottom, the sorted mCherry-low and -high populations (as indicated by red-outlined boxes and arrows) are shown directly after the sort, 14 days after the sort, and upon differentiation. Starting from the outermost contour, lines represent 7.5%, 22.5%, 37.5%, 52.5%, 67.5%, and 82.5% of the total events. (b) XGTC-XO ES cell clones after single-cell plating. (c) Expression levels of genes located in *Xic* as determined by RNA sequencing of XGTC-XO mCherry-low and -high populations. At the top is the location of genes along the X chromosome, and bars show log_2_(fragments per kilobase per million [FPKM] mCherry-low/FPKM mCherry-high) values. (d) Contour plots of data from FACS analysis showing EGFP and mCherry FIs for the XY Tsix-CHERRY ES line grown under serum-plus-LIF (top) (as shown in Fig. 4c) and 2i+LIF (bottom) conditions, prior to and at different time points after differentiation.

The striking bimodal mCherry distribution in XGTC-XO ES cells indicates that in similar proportions of cells, the *Tsix* promoter is either on or off. These two states switch very slowly, if at all. This is evident from the presence of two distinct populations considering the half-life of mCherry and the fact that recovery of the mixed population of mCherry- positive and -negative cells after FACS sorting of one of the populations does not occur within 2 weeks (Fig. 5a). Moreover, seeding of cells at a low density results in homogeneous colonies of either mCherry-negative or -positive cells (Fig. 5b). Also, differentiation of sorted mCherry-positive and -negative XGTC-XO ES cells did not lead to an increase in switching between states (Fig. 5a).

Staining for the differentiation marker CD31 and alkaline phosphatase activity, specific for undifferentiated embryonic stem cells, did not reveal differences in cell differentiation between the different cell populations (see Fig. S6a in the supplemental material). Also, bisulfite sequencing analysis of the *Tsix* promoter did not reveal differences between the mCherry-high and -low populations (see Fig. S6b in the supplemental material). To find the basis for the difference between the two populations, RNA sequencing was performed on FACS-sorted mCherry-positive and -negative XGTC-XO cells. This analysis indicated that both populations have highly similar expression profiles (Pearson correlation coefficient [*r*] = 0.9832) (see Fig. S6c in the supplemental material) and confirmed that the expression levels of the pluripotency factors were not different between the two cell populations (Pearson *r* = 0.999). Interestingly, close examination of expression levels of genes located in the Xic locus indicated several genes for which the expression level was correlated or anticorrelated with *Tsix* promoter-driven mCherry expression (Pearson *r* = 0.83) (Fig. 5c). These differences were most prominent for genes located within the Tsix TAD (Pearson *r* = 0.34) and suggest that the on-off switch of the *Tsix* promoter is based on distinct epigenetic states and/or the spatial conformation of the *Xic* locus.

**FIG 6:**
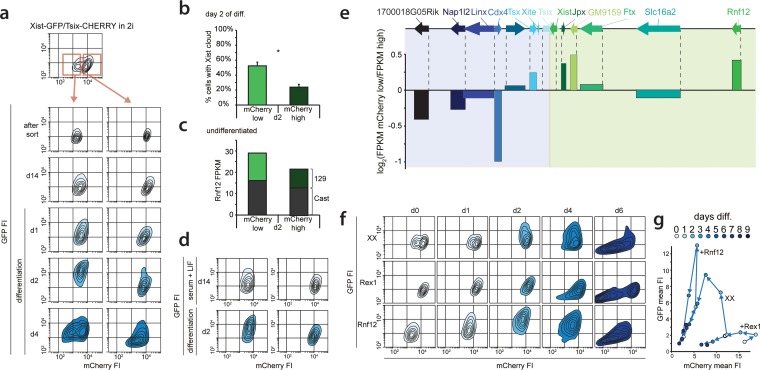
Two stable states of *Xic* predict XCI potential in XX cells. (a) Contour plots of data from FACS analysis showing EGFP and mCherry FIs for the Xist-GFP/Tsix-CHERRY ES cell line grown in 2i+LIF. The top panel depicts the original population with a bimodal mCherry distribution, and at the bottom, the sorted mCherry-low and -high populations (as indicated by the red-outlined boxes and arrows) are shown directly after the sort, 14 days after the sort, and upon differentiation. Starting from the outermost contour, lines represent 7.5%, 22.5%, 37.5%, 52.5%, 67.5%, and 82.5% of the total events. (b) Quantification of Xist RNA FISH in female Xist-GFP/Tsix-CHERRY cells at day 2 of differentiation after sorting of mCherry-low and -high populations. Error bars indicate 95% confidence intervals (*n* = 313 for mCherry-low and *n* = 305 for mCherry-high populations). The asterisk indicates a *P* value of <0.05 by a two-proportion *z* test. (c) Allele-specific RNA expression analysis by RNA sequencing. Shown are the FPKM values and allele-specific expression ratios (green, 129/Sv; shading, Cast). (d) Contour plots of 2i+LIF mCherry-high and -low Xist-GFP/Tsix-CHERRY ES cell populations 14 days after a change from 2i+LIF to serum-plus-LIF conditions (top) and 2 days later after the start of differentiation. (e) Expression levels of genes located in *Xic* as determined by RNA sequencing of 2i+LIF Xist-GFP/Tsix-CHERRY mCherry-low and -high populations. At the top is the location of genes along the X chromosome, and bars show log_2_(FPKM mCherry-low/FPKM mCherry-high) values. (f) Contour plots of data from FACS analysis showing EGFP and mCherry FIs at different time points of differentiation for Xist-GFP/Tsix-CHERRY (XX), Xist-GFP/Tsix-CHERRY+Rex1 (+Rex1), and Xist-GFP/Tsix-CHERRY+Rnf12 (+Rnf12) ES cells grown under 2i+LIF conditions. Starting from the outermost contour, lines represent 7.5%, 22.5%, 37.5%, 52.5%, 67.5%, and 82.5% of the total events. (g) Same as panel f but with mean FIs for EGFP and mCherry plotted.

Interestingly, under 2i+LIF conditions, which force ES cells to adopt a more naive state, the two distinct XY Tsix-mCherry and XGTC-XO ES cell populations became uniform (Fig. 5d and data not shown), suggesting that tissue culture conditions have a severe impact on the transcriptional states of *Xic*. Indeed, *Xist* qPCR analysis of wild-type 129/Sv-Cast/Eij female ES cells indicates that *Xist* is more repressed under 2i than under serum-plus-LIF conditions but that during ES cell differentiation, upregulation of *Xist* and skewing of XCI are not different under the two culture conditions (see Fig. S6d and e in the supplemental material). Nevertheless, 2i+LIF conditions impacted the transcriptional states of the Xic locus in female Xist-GFP/Tsix-CHERRY cells now displaying two separable mCherry populations, which were absent under serum-plus-LIF growth conditions (Fig. 6a). Again, after sorting of mCherry-low and -high cells, recovery of the mixed population of cells did not occur under 2i+LIF or differentiation conditions in a time frame of 2 weeks (Fig. 6a). Intriguingly, the mCherry-low population activates the Xist promoter-driven EGFP reporter much more strongly than does the mCherry-high population (Fig. 6a). This suggests that the potential to initiate XCI is determined by the state of *Xic* already before differentiation. Xist RNA FISH performed on these cells on day 2 of differentiation moreover indicates that the mutant and wild-type alleles coexist with a high probability in the same state, because cells from the mCherry-low population showed higher percentages of cloud formation (Fig. 6b). We also transferred Xist-GFP/Tsix-CHERRY ES cells to serum plus LIF to trigger a “primed” state (36). After 14 days of culturing under serum-plus-LIF conditions, mCherry levels stayed mostly stable, and preferential upregulation of Xist-GFP in mCherry-medium cells was still observed (Fig. 6d). Similar to our findings with XGTC-XO ES cells, RNA sequencing of undifferentiated mCherry-low and -high Xist-GFP/Tsix-CHERRY ES cells revealed marked differences between the two cell populations in genes located within the Xist and Tsix TADs (Fig. 6e). Allele-specific expression analysis of *Rnf12* showed increased *Rnf12* expression in mCherry-low cells but no preference for expression from the 129/Sv or Cast/Eij alleles, indicating that transcriptional states are synchronized between the wild-type and reporter alleles (Fig. 6c). Stabilization of these transcriptional states might be accomplished by feed-forward and feedback loops involving *Rnf12* and *Rex1*. To test this, we analyzed wild-type and *Rex1*- and *Rnf12*-transgenic Xist-GFP/Tsix-CHERRY ES cells cultured in 2i+LIF. FACS analysis revealed that *Rex1* overexpression forces cells to adopt the mCherry-high state, whereas *Rnf12* does the opposite, indicating that different transcriptional states are stabilized in *trans* by *trans*-acting factors (Fig. 6f and g). These findings argue that the on-off switch of the *Tsix* promoter is based on distinct epigenetic states and/or the spatial conformation of *Xic* and also explain the observed *Xist* promoter activation on both alleles in the mCherry-low population by increased levels of RNF12 (Fig. 6b and c). Our findings highlight the presence of differential epigenetic states, affected by extrinsic and intrinsic factors, capable of providing stable on-off switches for genes involved in XCI.

## DISCUSSION

In mouse, *Xist* and *Tsix* represent the key *cis*-regulatory players in the proper execution of XCI. This sense-antisense-transcribed gene couple fulfills antagonistic roles in the regulation of XCI, with the action of *Tsix* being restricted locally as a negative regulator of *Xist*, whereas *Xist* acts over large distances, silencing genes along the X chromosome. Our study confirms the repressive role of *Tsix* on *Xist* expression, although this effect appears most pronounced in undifferentiated ES cells. Xist upregulation is often interpreted to be the consequence of monoallelic Tsix downregulation (34, 37). Interestingly, our study indicates that *Xist* acts locally, facilitating the shutdown of *Tsix* not only on Xi but also on the future Xa chromosome, as we observed sustained *Tsix* expression in comparisons of three different Xist knockout ES cell lines with wild-type cells during ES cell differentiation. These findings indicate that *Xist* and *Tsix* are in constant interplay, as silencing of Tsix involves Xist-dependent and -independent mechanisms. Although this effect is likely mediated through Xist RNA-instructed local recruitment of chromatin remodeling complexes, we cannot exclude that a transcriptional interference mechanism may be involved.

Live-cell imaging of XX cells harboring Xist/Tsix fluorescent reporters also indicated that in the absence of overlapping sense-antisense transcription, the expression of *Xist* and *Tsix* is anticorrelated. Nevertheless, this anticorrelation is not strict, and we found *Xist* upregulation prior to *Tsix* downregulation and vice versa. This suggests a mechanism of stochastic expression of both genes, where the initiation of *Xist* expression is increased during differentiation until a level is reached that is sufficient for spreading in *cis*, leading to *Tsix* silencing and thereby providing a feed-forward loop facilitating further *Xist* transcription initiation, accumulation, and spreading.

**FIG 7:**
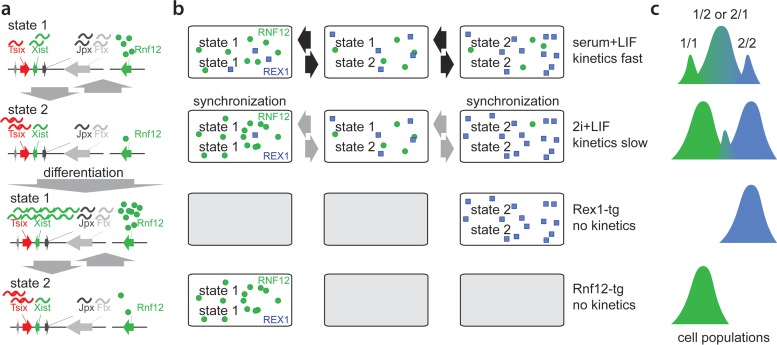
Model for dynamics of Xic transcriptional states. (a) Xic can adopt two distinct transcriptional states. State 1 is permissive whereas state 2 is refractive to Xist upregulation upon differentiation. (b) Under serum-plus-LIF conditions, female XX ES cells show rapid switching between different states, whereas under 2i+LIF conditions, state-switching dynamics are reduced, leading to synchronization of states. Rex and Rnf12 overexpression forces cells to adopt one single transcriptional state. (c) Relative quantity of alleles adopting distinct combinations of transcriptional states.

The present live-cell imaging studies indicate that the regulation of *Xist* and *Tsix* is rather stable in time and that *Xist* and *Tsix* expression in daughter cells preferably adopts the same fate. This might be related to *Xic* locus-intrinsic factors or to stable expression profiles of regulators of XCI. Studies involving XGTC-XO reporter cells grown under serum-plus-LIF conditions and XX Xist-GFP/Tsix-CHERRY reporter cells cultured in 2i-supplemented medium indicate that genes located within the *Xist* and *Tsix* TADs adopt different transcriptional fates, favoring the expression of a subset of genes. These distinct transcriptional fates might represent semistable states of the higher-order chromatin structure that can be propagated through many cell divisions and are different from previously reported X chromosome-wide cohesion differences (38). A recently developed polymer model predicted such different states of the higher-order chromatin structure (39). These transcriptional states are maintained independently of *Tsix* promoter methylation (see Fig. S6b in the supplemental material) and are likely independent of DNA methylation in general, which is nearly absent under 2i conditions (40). Switching between the different transcriptional states rarely occurs but is more frequently observed upon ES cell differentiation, which might be related to the reported increased chromatin dynamics during the early stage of ES cell differentiation (37), possibly provoked by changes in regulators of the XCI process. Under serum-plus-LIF conditions, no distinct subpopulations of XX ES cells are observed, suggesting that switching between states happens at a much higher frequency, with a shifted equilibrium constant, or that all cells adopt one and the same transcriptional state. Increased mobility of Xic has also been reported during early ES cell differentiation and might reflect the switching of transcriptional states described in this study (37). This does not necessarily mean that different transcriptional states as represented by the Tsix-mCherry-low and -high subpopulations are intrinsically stable. Rather, we favor a scenario in which the chromatin conformation fluctuates but exists preferentially in one conformation or the other (Fig. 7a). Our differentiation studies indicate that this transcriptional state in XX ES cells under serum conditions responds more homogeneously to differentiation cues than ES cells grown under 2i conditions. Nevertheless, in serum-plus-LIF-differentiated ES cells, we also observed cells that do not accumulate a PRC2 domain on Xi and continue to express the Xist-GFP reporter at high levels, suggesting that these cells are locked in an epigenetic state that does not allow the initiation of XCI on the wild-type X chromosome. The results obtained with 2i cells indicate that these transcriptional states can even predict the responsiveness of *Xic* to XCI regulators prior to the initiation of this process and that many cells do not initiate XCI at all. As Tsix-mCherry levels in serum plus LIF are equal to those of the Tsix-mCherry-high subpopulation under 2i conditions, which is more refractory to XCI initiation, this indicates that there are different transcriptional states that cannot be fully separated by Tsix levels only.

Interestingly, the present RNA FISH studies on sorted 2i populations indicate cross talk between the *Xic* loci with respect to this responsiveness, revealing significantly more cells initiating XCI on wild-type X in Tsix-mCherry-low than in Tsix-mCherry-high cells. This difference appears to be related to differences in the expression levels of activators and inhibitors of XCI, coordinated with the transcriptional state of *Xic*. A switch to a transcriptional state with a higher *Rnf12* transcription level on one allele results in increased RNF12-mediated turnover of REX1 and *Xist* activation. In general, several pluripotency factors act as repressors of *Rnf12* (13, 41), and also reduced REX1 levels may therefore facilitate switching to a transcriptional state with higher *Rnf12* expression levels on the second X chromosome, providing a feed-forward loop fixed in the transcriptional state (Fig. 7b and c). Our results might explain previously reported results obtained with differentiating ES cells grown under 2i conditions, which showed a high number of cells initiating XCI on both X chromosomes (42), and indicate that 2i culture conditions are suboptimal for studying the XCI process.

The reporter lines generated for this study nicely recapitulate XCI. Nevertheless, RNA and protein stability and differences in detection levels clearly affected our measurements. In our studies, we removed exon 1 completely, as a previous attempt to generate an Xist-EGFP reporter allele failed because the remaining *Xist* sequences prevented the nuclear export of the RNA (9). The removal of regulatory sequences and the introduction of the reporters themselves might therefore have impacted the regulation of *Xist* and *Tsix*. Previous work implicated RNF12 in the regulation of random XCI by the activation of *Xist* and repression of *Tsix*. ChIP analysis indicated two prominent REX1 binding peaks in both the *Xist* and *Tsix* intragenic regulatory elements. REX1-mediated repression of *Xist* involves competition of REX1 and YY1 binding for the same binding sites in the F-repeat region of Xist, with YY1 being an activator of *Xist* expression (21). Despite the removal of this F-repeat region from our reporter allele, we still found clear effects of *Rnf12* and *Rex1* overexpression on *Xist* regulation, indicating a role for alternative binding sites, such as those found in the *Xist* promoter, or indirect mechanisms to be instructive in *Xist* regulation. Our findings are supported by data from previous studies that also showed an effect of changes in *Rnf12* expression on luciferase reporters linked to the minimal *Xist* promoter (16, 20). Although our results suggest a prominent role for the RNF12-REX1 axis in the regulation of XCI, the effects on *Xist* and *Tsix* transcription were much more prominent in the absence of overlapping transcription, indicating that the activation of XCI requires a very balanced *cis*- and *trans*-acting environment for proper regulation. In addition, the severely reduced dynamics of Xist-GFP and Tsix-mCherry expression in XO reporter cell lines during ES cell differentiation also indicate that more X-linked factors are involved in the regulation of XCI. Interestingly, these factors also boost *Tsix* expression, which might be a requirement for the proper execution of a mutually exclusive XCI process, providing a stable binary switch. XCI activators therefore seem to act at two different levels, first by bringing *Xic* to a transcriptional state that allows the proper execution of XCI and second by providing sufficient *Xist* promoter activity through direct and indirect mechanisms.

## Supplementary Material

Supplemental material
