# Supplementary material for: Xist and Tsix Transcription Dynamics Is Regulated by the X-to-Autosome Ratio and Semistable Transcriptional States
Source: Mol Cell Biol. 2016 Oct 13;36(21):2656–67. doi: 10.1128/MCB.00183-16 (PMC5064214; doi:10.1128/MCB.00183-16)
Supplement: Supplemental material [file supp_36_21_2656__index.html]

Supplemental material 

# *Xist* and *Tsix* Transcription Dynamics Is Regulated by the X-to-Autosome Ratio and Semistable Transcriptional States

## Supplemental material

- Supplemental file 1 -

  Fig. S1 (Targeting strategy)

  PDF, 55K
- Supplemental file 2 -

  Fig. S2 (Targeting of cell lines)

  PDF, 194K
- Supplemental file 3 -

  Fig. S3 (Behavior of wild-type and mutant alleles of *Xist* and *Tsix*)

  PDF, 24K
- Supplemental file 4 -

  Fig. S4 (Life cell imaging of reporter lines)

  PDF, 266K
- Supplemental file 5 -

  Fig. S5 (Generation and analysis of Rnf12 and Rex1 transgenic and mutant and XO ES cell lines)

  PDF, 185K
- Supplemental file 6 -

  Fig. S6 (RNA expression analysis of XGTC‐XO, mCherry low and high subpopulations)

  PDF, 85K
- Supplemental file 7 -

  Supplemental figure legends and table caption

  PDF, 165K
- Supplemental file 8 -

  Table S1 (Primers)

  PDF, 76K
