# Supplementary figures and images for: Xist and Tsix Transcription Dynamics Is Regulated by the X-to-Autosome Ratio and Semistable Transcriptional States
Source: Mol Cell Biol. 2016 Oct 13;36(21):2656–67. doi: 10.1128/MCB.00183-16 (PMC5064214; doi:10.1128/MCB.00183-16)

a

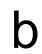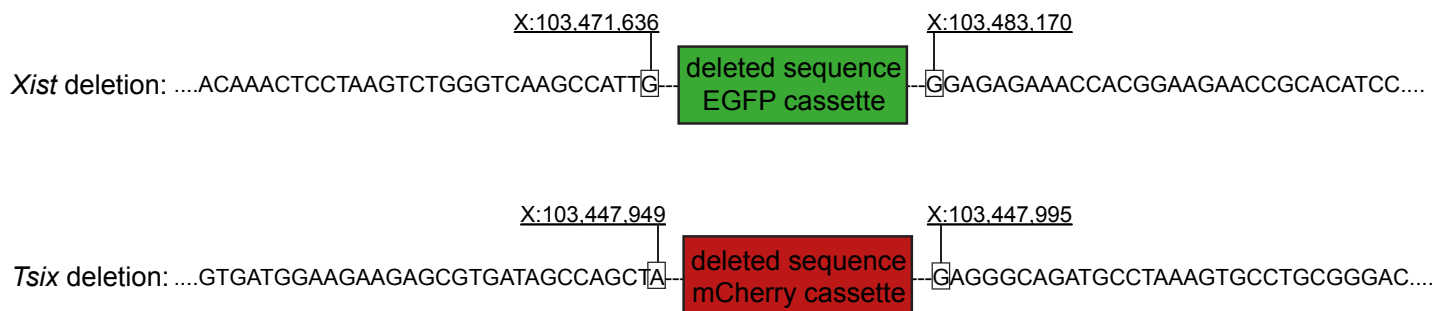

Supplement: Supplemental material [file MCB.00183-16_zmb999101334so1.pdf]

# Supplementary Figure 2

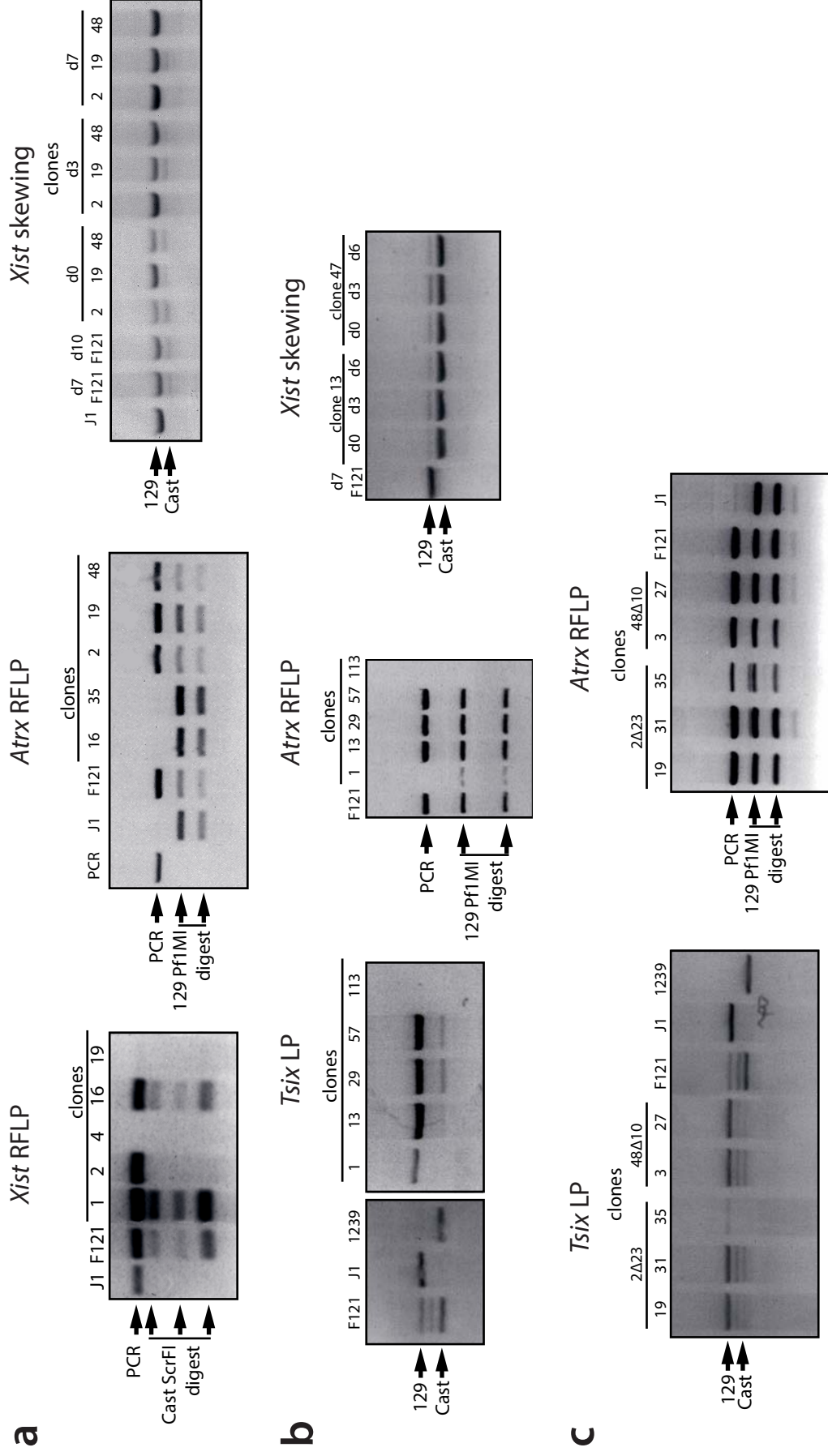

Supplement: Supplemental material [file MCB.00183-16_zmb999101334so2.pdf]

Supplementary Figure 3

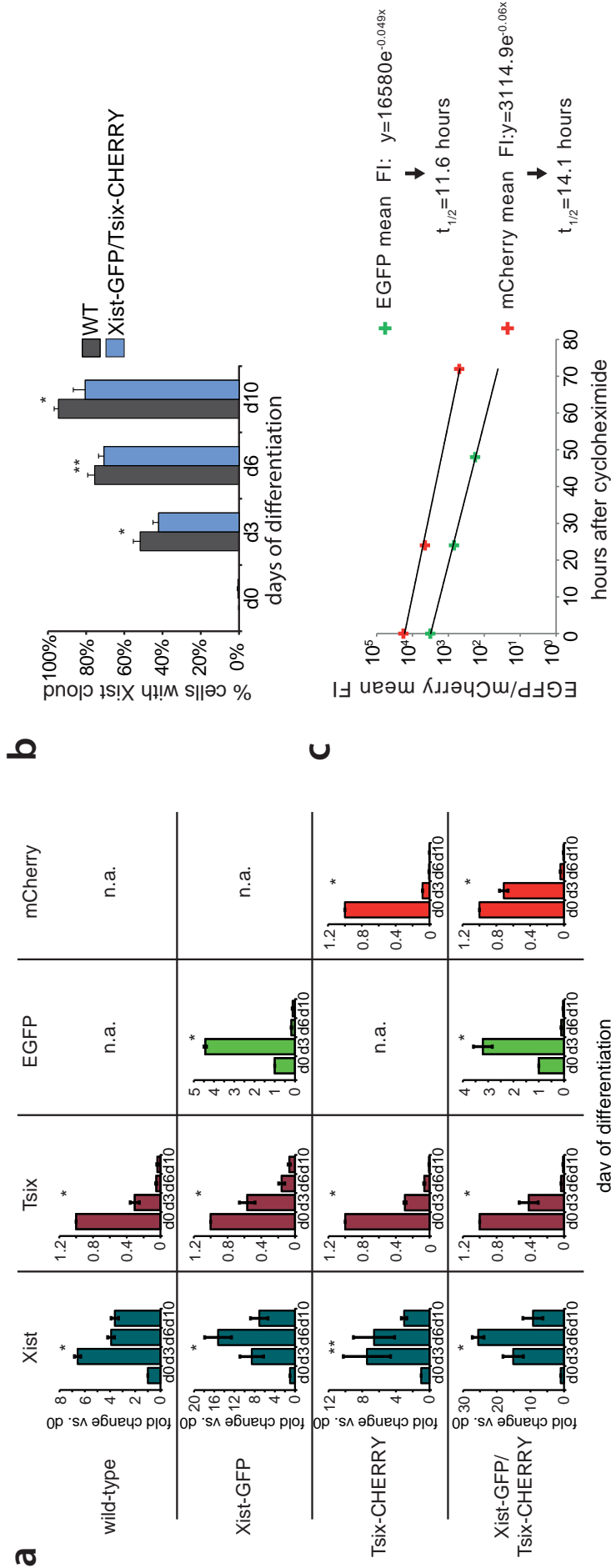

Supplement: Supplemental material [file MCB.00183-16_zmb999101334so3.pdf]

# Supplementary Figure 4

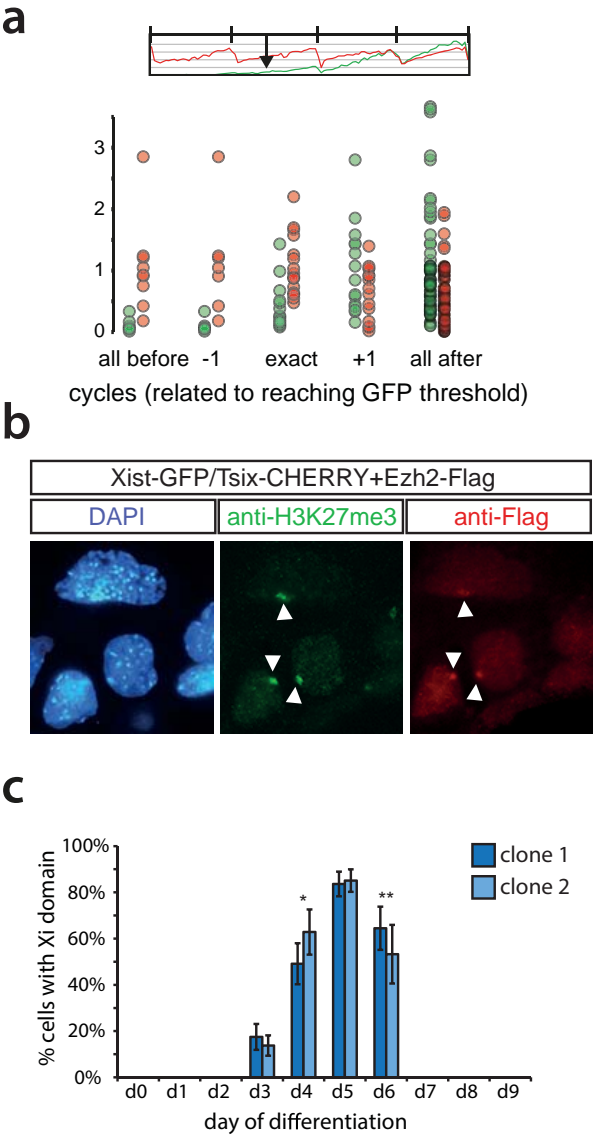

Supplement: Supplemental material [file MCB.00183-16_zmb999101334so4.pdf]

# Supplementary Figure 5

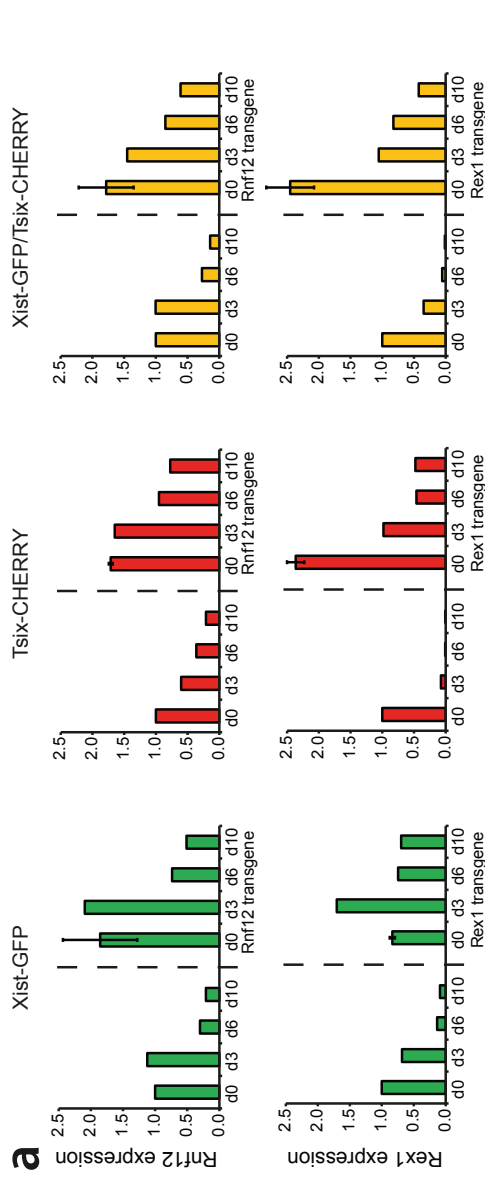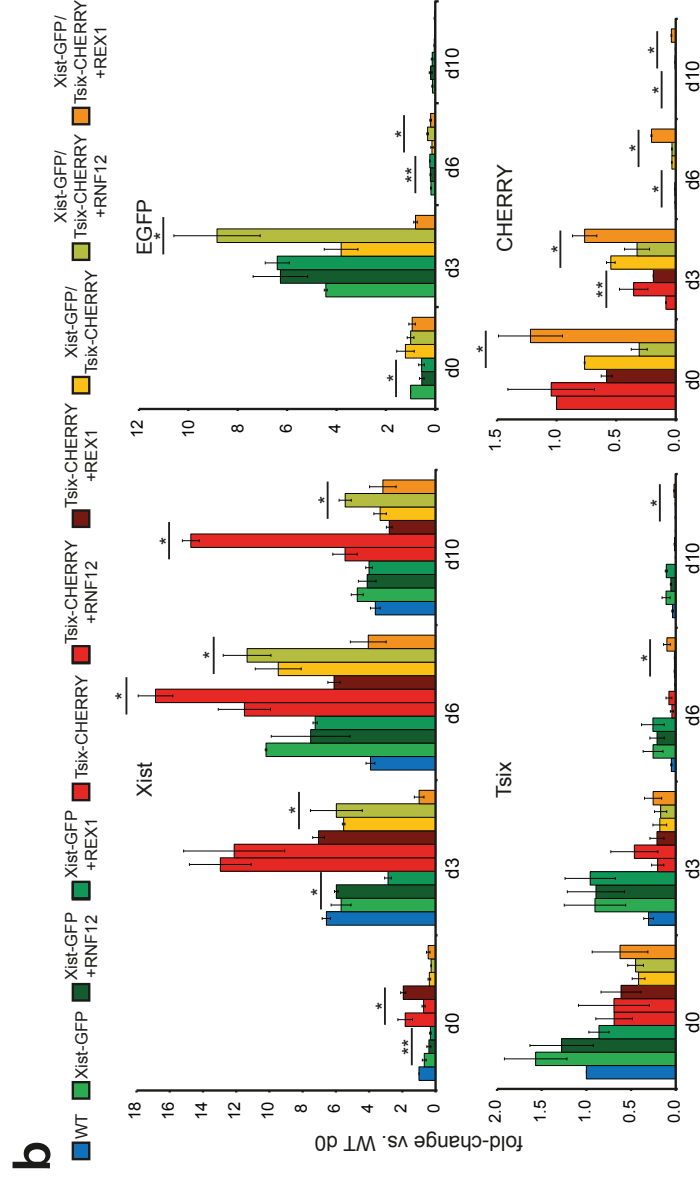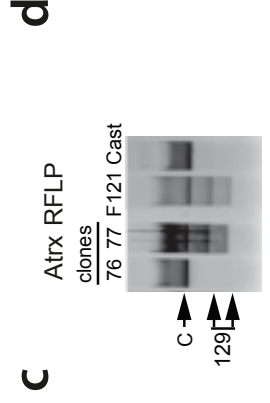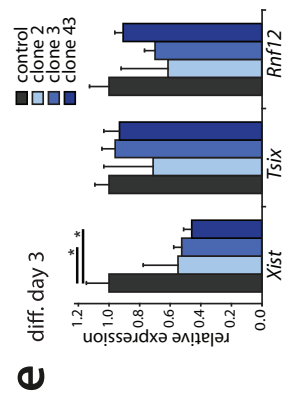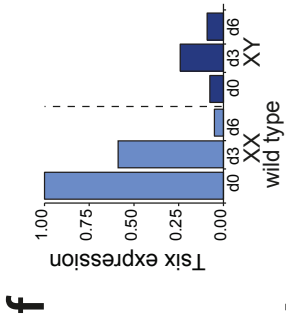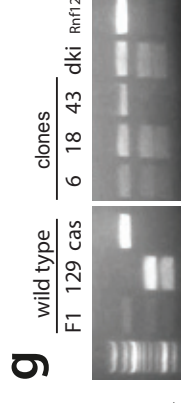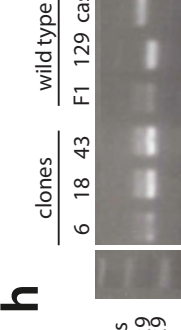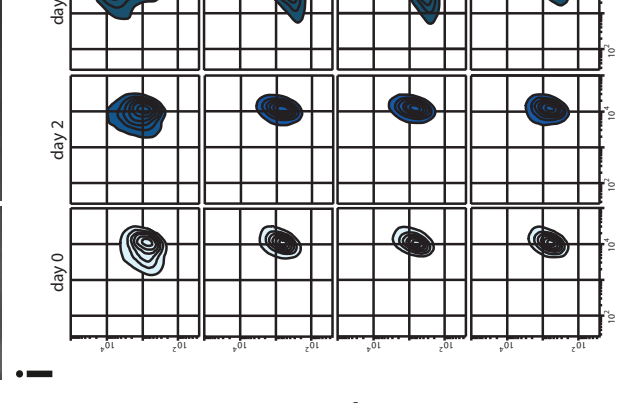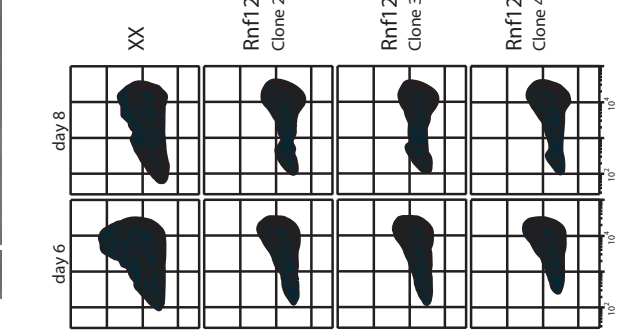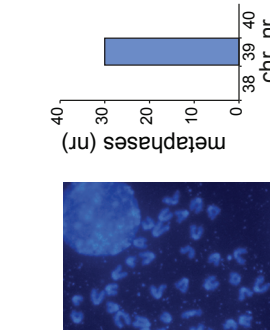

Supplement: Supplemental material [file MCB.00183-16_zmb999101334so5.pdf]

## Supplementary Figure 6

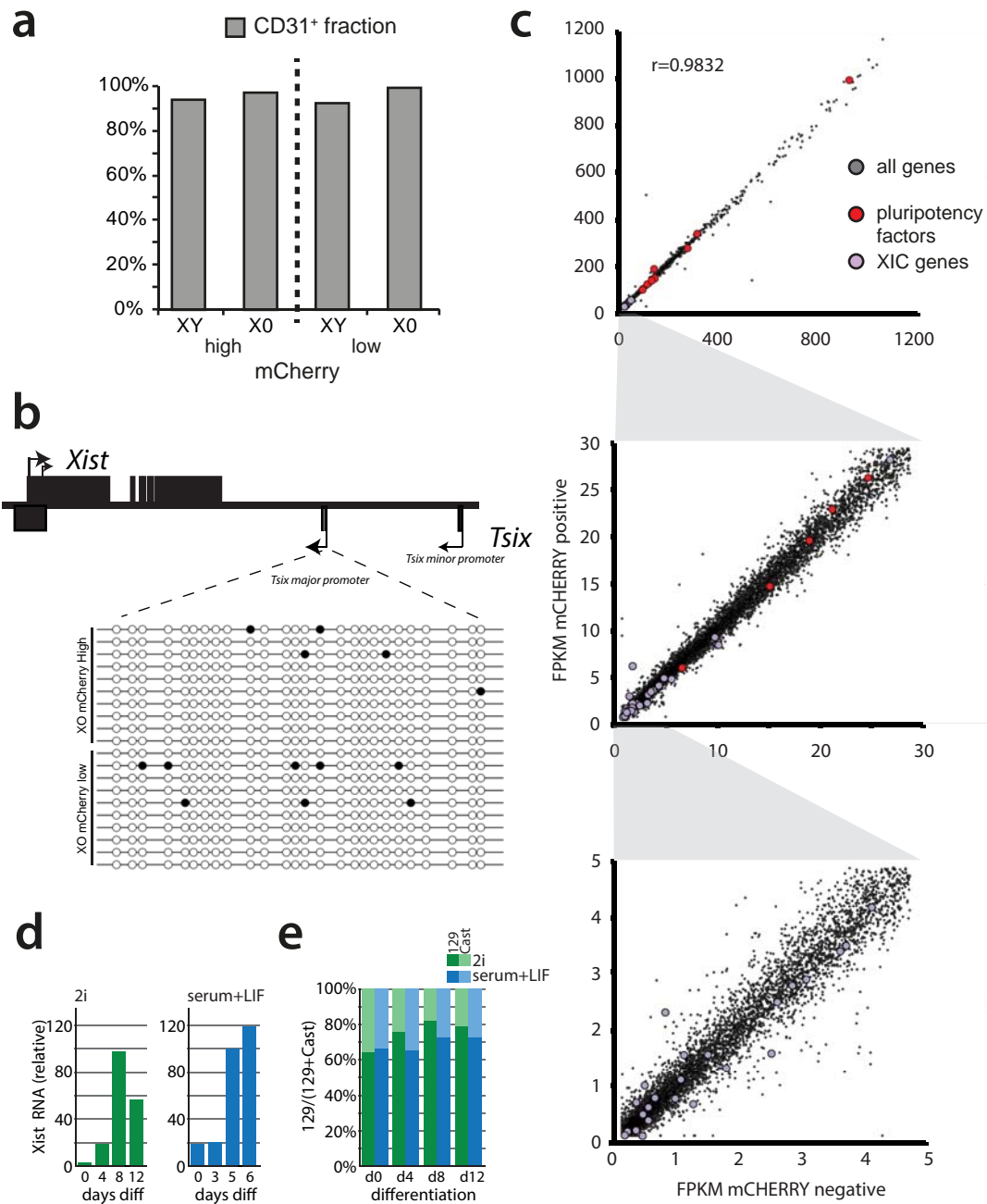

Supplement: Supplemental material [file MCB.00183-16_zmb999101334so6.pdf]
