# Supplementary material for: Xist and Tsix Transcription Dynamics Is Regulated by the X-to-Autosome Ratio and Semistable Transcriptional States
Source: Mol Cell Biol. 2016 Oct 13;36(21):2656–67. doi: 10.1128/MCB.00183-16 (PMC5064214; doi:10.1128/MCB.00183-16)
Supplement: Supplemental material [file MCB.00183-16_zmb999101334so7.pdf]

Supplementary Figure legends

Supplementary Figure 1. Targeting strategy.

Targeting scheme for generation of Xist-GFP, Tsix-CHERRY and Xist-GFP/Tsix-CHERRY murine ES cell lines. BAC name, location and relative size are indicated on top of panel. Lower part of panel depicts mCherry and EGFP targeting cassettes, exon-intron structure of Tsix (grey) and Xist (black), and position of genotyping (black arrowheads) and phenotyping (red arrowheads) primers. Primer numbers are given as in Supplementary Table 1. Polymorphisms used for screening of correctly targeted clones are a length polymorphism (LP) in the DXPas34 region and an ScrFI restriction fragment length polymorphism (RFLP) in exon 1 of Xist.

Supplementary Figure 2. Targeting of Cell Lines.

(a) Targeting of EGFP to the *Xist* locus in female wild-type 129/Sv-Cast/Ei ES cell line. Left panel shows PCR amplification and ScrFI RFLP digest of PCR product to identify clones with a correctly targeted Cast/Ei *Xist* allele. Correct targeting of EGFP-cassette to Cast/Ei allele results in loss of Cast/Ei-specific restriction products, as shown for clone 2. Arrows on left indicate size of PCR product and size of ScrFI restriction fragments. J1 is a 129/Sv control, F121 the polymorphic 129/Sv-Cast/Ei mother cell line. Center panel shows PCR amplification and Pf1MI digest of an X-linked PCR product from the *Atrx* gene to verify presence of two X chromosomes. Arrows on left indicate size of PCR product and size of Pf1MI restriction fragments. J1 is a 129/Sv control, F121 the polymorphic 129/Sv-Cast/Ei mother cell line. For example, clones 16 and 35 had lost the Cast X chromosome. Right Panel shows PCR on cDNA over an *Xist* length polymorphism, demonstrating that in clone 2 only 129/Sv *Xist is* expressed upon differentiation (lower Cast band represents transcription read through only detectable in undifferentiated samples). Arrows on left indicate size of 129/Sv and Cast/Ei PCR products.

(b) Targeting of mCherry to the *Tsix* locus in female wild-type 129/Sv-Cast/Ei ES cell line. Left panel shows PCR amplification of an Tsix length polymorphis on genomic DNA to identify clones with a correctly targeted Cast/Ei *Tsix* allele. Correct targeting of mCherry-cassette to Cast/Ei allele results in loss of Cast/Ei-specific band, as shown for clone 13. Arrows on left indicate size of PCR product for 129/Sv and Cast/Ei alleles. J1 is a 129/Sv control, 1239 is a Cast/Ei control and F121 is the polymorphic 129/Sv-Cast/Ei mother cell line. Center panel shows PCR amplification and Pf1MI digest on *Atrx* as in (A).

Right panel shows PCR on cDNA over an *Xist* length polymorphism, demonstrating that in clone 13 *Xist* skewing is reversed and *Xist* is primarily expressed from Cas/Ei allele.

(c) Targeting of mCherry to the *Tsix* locus in Xist-GFP ES cell line. Left and center panel as in (b), showing correct targeting in clone 2Δ23.

Supplementary Figure 3. Behavior of Wild Type and Mutant Alleles of *Xist* and *Tsix.*

(a) Expression analysis of *Xist*, *Tsix*, EGFP, mCherry expression levels at different time points of differentiation by quantitative RT-PCR. Quantification is depicted as fold change as compared to undifferentiated cells. Of note, in wild type cells Xist and Tsix levels arise from both the future Xa and Xi; in Xist-GFP Xist arises from future Xi, Tsix from both future Xa and Xi and EGFP from future Xa; in Tsix-CHERRY Xist arises from both future Xa and Xi, Tsix from future Xa and mCherrry from future Xi; in Xist-GFP/Tsix-CHERRY Xist and Tsix arise from future Xi, while EGFP and mCherry arise from future Xa. Error bars represent SD of two or three independent experiments.

(b) Quantification of Xist RNA FISH in differentiating wild type and Xist-GFP/Tsix-CHERRY cells. Error bars indicate 95% confidence interval, $n > 100$ for day 0, $n > 350$ for day 3 and 6, $n > 150$ for day 10.

(c) Determination of half-life of EGFP and mCherry reporter proteins by cycloheximide chase and FACS analysis of mean FI values for EGFP and mCherry. Xist-GFP and Tsix-CHERRY cells were treated with 100μg/ml cycloheximide (Sigma) to stop protein synthesis and decay of fluorescent proteins was monitored over time. Values were fitted to a first order decay function to estimate the degradation rate constant k and half-life was calculated as: $t1/2=\ln(2)/k$.

Asterisks indicate $P < 0.05$ (*) or $P < 0.1$(**) by single-factor analysis of variance (a) or two-proportion z-test (b).

Supplementary Figure 4. Life cell imaging of reporter lines.

(a) Linear regression of FI over time for each cell cycle was performed. Slope of linear regression as a proxy for promoter activity is plotted. Bins are chosen according to time point of *Xist* promoter activation. Threshold for *Xist* activation was set at 3.29 SDs (corresponding to 99.9% within confidence

interval) of background mean EGFP FI measured within the first six hours of time-lapse experiment. Bins as depicted in cartoon on top of panel were chosen as follows: The exact cell cycle in which EGFP FI threshold is reached (exact), one cell cycle before or after threshold is reached (-1,+1) and all cell cycles before or after threshold is reached (all before, all after).

(b) Immunofluorescence staining for H3K27me3 and Flag in Xist-GFP/Tsix-CHERRY line at day 3 of differentiation. White arrowheads indicate Xi domain as identified by H3K27me3 and Ezh2-Flag staining.

(c) Quantification of Xist-GFP/Tsix-CHERRY cells showing transient enrichment of Ezh2-Flag on the Xi during differentiation determined by direct detection of fluorescence. Two different transgenic clones are shown. Error bars indicate 95% confidence interval, n $>$ 150 for all time points showing Xi domains, n=100 for all time points without Xi domains. Asterisks indicate $P < 0.05$ (*) or $P < 0.1$(**) by two-proportion z-test.

Supplementary Figure 5. Generation and analysis of Rnf12 and Rex1 transgenic and mutant and XO ES cell lines.

(a) Expression analysis of *Rnf12* and *Rex1* at different time points of differentiation by quantitative RT-PCR. Xist-GFP, Tsix-CHERRY and Xist-GFP/Tsix-CHERRY lines plus the corresponding Rnf12 and Rex1 transgenic lines are shown. Quantification is depicted as fold change as compared to undifferentiated cells without Rnf12 or Rex1 transgenes. Error bars represent SD of two independent experiments.

(b) Expression analysis of *Xist*, *Tsix*, EGFP, mCherry expression levels at different time points of differentiation by quantitative RT-PCR. Xist-GFP, Tsix-CHERRY and Xist-GFP/Tsix-CHERRY lines plus the corresponding Rnf12 and Rex1 transgenic lines are shown. Quantification is depicted as fold change as compared to undifferentiated cells without Rnf12 or Rex1 transgenes. Error bars represent SD of two independent experiments, asterisks indicate $P < 0.05$ (*) or $P < 0.1$(**) by single-factor analysis of variance for RNF12/REX1 transgenic cell lines and their respective mother cell lines.

(c) Screen to identify loss of wild type X chromosome in subclones of Xist-GFP/Tsix-CHERRY by utilizing an X-linked RFLP. PCR amplification and Pf1MI digest of an X-linked PCR product from the *Atrx* gene is shown. Arrows on left indicate size of PCR product and size of Pf1MI restriction fragments. F121 is the

polymorphic 129/Sv-Cast/Ei mother cell line, Cast is pure Cast/Ei control. Four of 384 clones showed loss of an X chromosome including clone 76 which lost the wild type 129/Sv X chromosome.

(d) Karyotype analysis of XGTC-XO ES cells prior to FACS analysis.

(e) Xist, Tsix and Rnf12 q-PCR expression analysis comparing day 3 differentiated control and three experimental Rnf12$^{+/-}$ ES cell lines. Asterisks indicate $P < 0.05$ (*) by Student's t-Test.

(f) Targeting of Rnf12 in the Xist-GFP/Tsix-CHERRY ES cell line. Shown is PCR amplification of an RFLP on genomic DNA to identify clones with a correctly targeted Rnf12 allele. Correct targeting results in loss of the 129/Sv allele. Arrows on left indicate size of PCR product for 129/Sv and Cast/Ei alleles. Shown are 129/Sv-Cast/Ei (F1), 129/Sv (129) and Cast/Eij (cas) controls, and the starting Xist-GFP/Tsix-CHERRY (dki) and Rnf12$^{+/-}$ ES cell lines.

(g) PCR amplification of DXMit65 length polymorphism on genomic DNA, to confirm presence of two X chromosomes.

(h) Contour plots of FACS analysis showing EGFP and mCherry FI for the Xist-GFP/Tsix-CHERRY control and three Rnf12$^{+/-}$ ES cell lines at different time points of differentiation. Starting from outermost contour, lines represent 7.5%, 22.5%, 37.5%, 52.5%, 67.5%, 82.5% of total events.

(i) Expression analysis of *Tsix* at different time points of differentiation by quantitative RT-PCR. Wild type female XX and male XY cell lines are shown. Quantification is depicted as fold change as compared to undifferentiated female cells.

Supplementary Figure 6. RNA expression analysis of XGTC-XO, mCherry low and high subpopulations.

(a) FACS analysis of mCherry levels and pluripotency marker CD31 in XY Tsix-CHERRY (XY) and XGTC-XO (XO). Percentage of CD31^{+} cells is shown for mCherry low and high populations, indicating that there is no difference in pluripotent state between the mCherry low and high populations.

(b) Bisulfite sequencing analysis of the *Tsix* major promoter region in XO mCherry low and high populations (empty and filled circles depict unmethylated and methylated CpG sequences respectively).

(c) RNA sequencing of XGTC-XO mCherry low and high populations. FPKM values for all genes are plotted, red dots are pluripotency factors, blue dots genes located in the *Xic*. From top to bottom zoom in is depicted as indicated on axes. Pearson correlation coefficient r=0.9832.

(d) *Xist* qPCR expression analysis at different time points during ES cell differentiation of wild type 129/Sv:Cast ES cells cultured in serum+LIF and 2i conditions.

(e) Allele specific expression analysis of *Xist* during ES cell differentiation indicates skewing of *Xist* expression throughout the XCI process.

Supplementary Table 1. Primers used in this study as listed in the Materials and Methods section.
