# Supplementary material for: Xist and Tsix Transcription Dynamics Is Regulated by the X-to-Autosome Ratio and Semistable Transcriptional States
Source: Mol Cell Biol. 2016 Oct 13;36(21):2656–67. doi: 10.1128/MCB.00183-16 (PMC5064214; doi:10.1128/MCB.00183-16)
Supplement: Supplemental material [file MCB.00183-16_zmb999101334so8.pdf]

#	SEQUENCE	DESCRIPTION
1	AGGTACCTCCCAAGGTATGGAGTCACC	Forward primer 5' targeting arm for *Xist*
2	TACCGGTAGGAGAGAAACCACGGAAGAA	Reverse primer 5' targeting arm for *Xist*
5	TAGTACTCAATGGCTTGACCCAGACTT	Forward primer 3' targeting arm for *Xist*
6	TAGTACTGTGCCAGAAGAGGGAGTCAG	Reverse primer 3' targeting arm for *Xist*;<br>Reverse primer ScrFI RFLP in *Xist*
20	GCTGGTTCGTCTATCTTGTGG	Forward primer ScrFI RFLP in *Xist*
25	CTTTGGTCTCTGGGTTTCCA	Forward primer 5' targeting arm for *Tsix*
27	TACCGGTAGCTGGCTATCACGCTCTTC	Reverse primer 5' targeting arm for *Tsix*
29	GAGGGCAGATGCCTAAAGTG	Forward primer 3' targeting arm for *Tsix*
30	CGCAGGCATTTTACCTTCAT	Reverse primer 3' targeting arm for *Tsix*
36	AGTGCAGCGCTTGTGTCA	Forward primer *Tsix* length polymorphism, for DNA
41	TATTACCCACGCCAGGCTTA	Reverse primer *Tsix* length polymorphism, for DNA
68	TCCCCAATTA AAGGTGTTGA	Forward primer Pf1MI RFLP in *Atrx*
69	AATTCACGTTCTCCTCTTTCACT	Reverse primer Pf1MI RFLP in *Atrx*
106	AGGGCATCGACTTCAAGGAG	Forward primer EGFP expression
107	CACCTTGATGCCGTTCTTCTG	Reverse primer EGFP expression
108	CCCGTAATGCAGAAGAAGACC	Forward primer mCherry expression
109	CTTCAGCCTCTGCTTGATCTC	Reverse primer mCherry expression
137	GTGATGGAAGAAGAGCGTGA	Forward primer *Tsix* expression
138	GCTGCTTGGCAATCACTTTA	Reverse primer *Tsix* expression
157	AACCCTAAGGCCAACCGTGAAAAG	Forward primer *Actb* expression
158	CATGGCTGGGGTGTTGAAGGTCTC	Reverse primer *Actb* expression
159	GGATCCTGCTTGAACTACTGC	Forward primer *Xist* expression
160	CAGGCAATCCTT CTTCTTGAG	Reverse primer *Xist* expression
1445	ACTGGGTCTTCAGCGTGA	Forward primer *Xist* length polymorphism exon 6-7, for RNA
1446	GCAACAACGAATTAGACAACAC	Reverse primer *Xist* length polymorphism exon 6-7, for RNA
